# Barriers and applied activity, quality of life and self-efficacy in prostate cancer survivors 1 year after completing radiotherapy

**DOI:** 10.1007/s00520-023-07729-z

**Published:** 2023-04-20

**Authors:** Javier Martín-Núñez, Marta Linares-Moya, Andrés Calvache-Mateo, Antonio Lazo-Prados, Alejandro Heredia-Ciuró, Laura López-López, Marie Carmen Valenza

**Affiliations:** 1 Department of Physiotherapy, Faculty of Health Sciences, University of Granada, Av. De La Ilustración, 60, 18016 Granada, Spain; 2 Oncological Radiotherapy Service of the “Hospital PTS”, Clínico San Cecilio University Hospital, Granada, Spain

**Keywords:** Prostate cancer, Radiotherapy, Physical activity, Self-efficacy, Quality of life

## Abstract

**Purpose:**

The aims of the study were to assess self-reported physical activity (PA) levels, barriers to PA, quality of life and self-efficacy to manage chronic disease of prostate cancer survivor 1 year after radiotherapy treatment.

**Methods:**

A cross-sectional case–control study was performed. Prostate cancer survivor patients treated with radiotherapy were recruited from the Radiation Oncology Service of the “Complejo Hospitalario Universitario” (Granada) and compared with age-matched healthy men. Outcomes included were perception of benefits for physical activity and potential barriers (Exercise Benefits/Barriers Scale), physical activity levels assessed by the International Physical Activity Questionnaire (IPAQ), quality of life (EuroQol five-dimension three-levels) and self-efficacy (Self-Efficacy to Manage Chronic Disease).

**Results:**

A total of 120 patients were included in our study. Significant differences were found between groups with worse results for the prostate cancer patient group in the variable perception of the benefit of physical activity, potential barriers, and physical activity. Regarding quality of life and self-efficacy, significant differences were also observed between groups with a greater score in the control group.

**Conclusion:**

In conclusion, the results of this study reveal that self-reported PA levels, as measured using the IPAQ, were low in prostate cancer survivors after treatment. Results also showed worse perception of benefits for PA and potential barriers by the cancer survivors. Similarly, the quality of life and self-efficacy to manage chronic disease of prostate cancer survivors was lower.

## Introduction

The constant improvement of cancer treatments as well as diagnostic methods has significantly increased the life expectancy of cancer patients. Survival of a cancer diagnosis is expected to be greater than 60% [1, 2], which is a major health challenge [3]. A considerable number of cancer patients experience comorbidities and symptoms secondary to cancer, even years after initial treatment [4]. Patients who survive cancer treatment often experience persistent side effects such as sleep disturbances [5], pain [6] and fatigue [7]. In addition, they experience other comorbidities such as diabetes, osteoporosis, cardiovascular disease, functional impairment and ultimately an increased risk of new primary cancers [8].

Prostate cancer is a significant health burden expected to increase over the next years due to the recent survival data [9]. Despite earlier detection, prostate cancer patients use to receive treatment and exhibit side effects of therapy during long-term survival [10].

A relevant aspect of cancer survivorship is related to lifestyle behaviours, with a key role in physical activity [11–13]. According to previous studies, physical activity can improve survival, the risk of cancer recurrence and the quality of life of cancer survivors [14–16]. Most survivors do not engage in regular physical activity, and less than 30% achieve minimum levels, despite the benefits of physical activity [17, 18]. Different studies have explored factors related to physical activity after a cancer diagnosis, finding education, age, body mass index, occupation and receiving specific cancer therapies among the most important [19, 20].

Results obtained in various meta-analyses have shown an inverse association between amounts of physical activity after diagnosis and cancer-specific mortality in prostate cancer survivors [21–23]. Those systematic reviews indicate that the highest levels of total, recreational, non-sedentary occupational, and vigorous physical activity, including higher metabolic equivalent (MET) hours per week, were significantly related to reduced risk for all-cause mortality.

Despite the volume of evidence indicating the benefits of regular physical activity for health and functioning [23, 24], people with cancer are far less likely to engage in physically active lifestyles, and the enrolment of these patients in physical activity (PA) programs remains unsuccessful [18, 25]. Little is known about why the majority of people with cancer fail to integrate regular physical activity into their lifestyle [26]. It has been suggested that an understanding of potential barriers that affect participation by cancer patients could provide important information necessary for developing interventions that have a greater likelihood of success [27]. Previous research has identified different aspects related to physical activity levels such as pain, cancer treatment-related side effects, fatigue, motivation, comorbid medical conditions and time [20, 28, 29]. Despite this, the literature referring to prostate cancer survivors examining barriers to physical activity [20, 30, 31] is very limited and has not explored the specific profile of long-term patients after radiotherapy.

The objectives of our study were to (i) measure self-reported PA levels, (ii) assess perceived barriers to PA, (iii) and determine quality of life and self-efficacy to manage chronic disease of prostate cancer survivor 1 year after radiotherapy treatment. All these factors are determinants in improving the enrolment of prostate cancer survivors in PA programs.

## Methods

### Design and ethics

A cross-sectional study was conducted between January 2022 and April 2022. Before being included in the study, patients received detailed information about the study goals and procedure and gave their informed consent to participate. The study was approved by a local committee on research ethics.

### Population

Prostate cancer survivor patients treated with radiotherapy were recruited from the Radiation Oncology Service of the “Complejo Hospitalario Universitario” (Granada). The eligibility criteria for the prostate cancer patients included histologically documented prostate cancer, 1 year after completion of radiotherapy treatment and no on-going cancer treatment. The control cohort included aged-matched healthy men with similar body weight and height, with no previous history of cancer. Control participants were recruited by word-of-mouth and were excluded if they exhibited any history of cancer. Matching for aged and BMI was achieved by individually selecting the control subject with the closest available match for age and BMI to the prostate cancer survivor patients.

Case and control participants were excluded if they had one of these conditions: under 18 years of age, neurologic pathologies limiting voluntary mobility, orthopaedic and cardiovascular pathologies, learning disability or if telephone contact was inappropriate due to dementia, or other cognitive or communication impairment.

An a priori power analysis based on a pilot study (unpublished) of 10 subjects (effect size of 0.80) was performed with the G*Power 3.1.9.2 software (3.1.9.2v; Statistical Power Analyses for Windows, Universität Düsseldorf, Germany) resulting in a sample size of 104 patients (52 per group) and a statistical power of 90%. Considering a hypothetical dropout rate of 10%, 58 patients were needed in each group. Recruitment ended when the required sample size was reached for each group.

### Measurements

Participants were assessed by telephone always by the same investigators previously trained. An initial assessment interview was conducted to confirm that the patients met the inclusion criteria. Data regarding comorbidities, anthropometric data, prostate cancer characteristics and cancer treatment were obtained from the medical history. The Charlson index was used to assess comorbidities [32] which has been validated in several disorders and is one of the most widely used scoring systems for assessing comorbidities.

The participant’s perception of benefits for physical activity and potential barriers was measured with the Spanish version of Exercise Benefits/Barriers Scale (EBBS) [33]. The scale includes 43 items separated into two subscales: 14 items refer to barriers and 29 items refer to benefits [34]. The scale is designed based on a 4-point Likert scale: strongly disagree (1), disagree (2), agree (3), strongly agree (4). For the benefits subscale, the answer range varies between 29 and 116 and the higher the score, the more positively the individual perceives exercise. For the barriers subscale, the answer range varies between 14 and 56, and the higher the score, the more negatively the individual perceives exercise. When all items are summed to obtain a total score, the barrier to exercise subscale items are reverse scored. In contrast, when only the barriers to exercise subscale is calculated, no inverse score is applied to these items [35]. When the total sum of barriers and benefits is summed, the score can range from 43 to 172. In this case, the higher the score, the more positively the individual perceives exercise [36].

The physical activity levels were evaluated with the Spanish version of the International Physical Activity Questionnaire (IPAQ) [37]. It has been validated and previously used in cancer patients. This questionnaire was designed to quantify physical activity in transportation, household chores, work and leisure time. Subjects are asked to report both the frequency and duration of activities performed during the last week divided into three categories: walking, moderate activities and vigorous activities. Activity is calculated as the total time spent in the three activity categories. A metabolic equivalent (MET) is used to weight the total task time, resulting in an estimate of activity that is expressed as MET-min/week and adjusted for body weight [38].

To assess quality of life, the five-dimension, three-level EuroQol (EQ-5D-3L) was used in its Spanish version, which is divided into two distinct sections [39, 40]. The first section is divided into 5 items related to mobility, usual activities, self-care, anxiety/depression and pain/discomfort. Each of the items has three response levels corresponding to “no problems”, “some problems” or “extreme problems”. The second part of the scale consists of a visual analogue scale (VAS) in which the respondents must self-assess their current health status by assigning a score between 0 (worst imaginable health status) and 100 (best imaginable health status). The EQ-5D-3L has previously been used in prostate cancer patients [41].

The Spanish version of the scale to measure Self-Efficacy to Manage Chronic Disease (SEMCD-S) was used to assess self-efficacy [42]. The scale consists of 4 items which are answered with a score from 1 (no confidence) to 10 (total confidence). To obtain the result of the scale, the mean of the 4 items is calculated. If more than one of the items is not answered, the final score cannot be calculated. The SEMCD-S has been used previously in cancer patients [43].

### Data analysis

Statistical analysis was performed with IBM SPSS Statistics software for Windows, Version 20.0 (IBM Corp. Released 2011; Armonk, NY: IBM Corp). Descriptive statistics were used to describe sample baseline characteristics. Categorical variables are presented as a percentage (%), and continuous variables are presented as the mean ± standard deviation. The Kolmogorov–Smirnov test was performed to assess continuous data normality, prior to statistical analysis. For data with a normal distribution, Student’s *t* test was performed, a Wilcoxon test to non-parametric variables and a χ^2^ test for nominal variables. The statistical analysis was conducted at a 95% confidence level. A *p* value *p* < 0.05 was considered statistically significant.

## Results

**Fig. 1:**
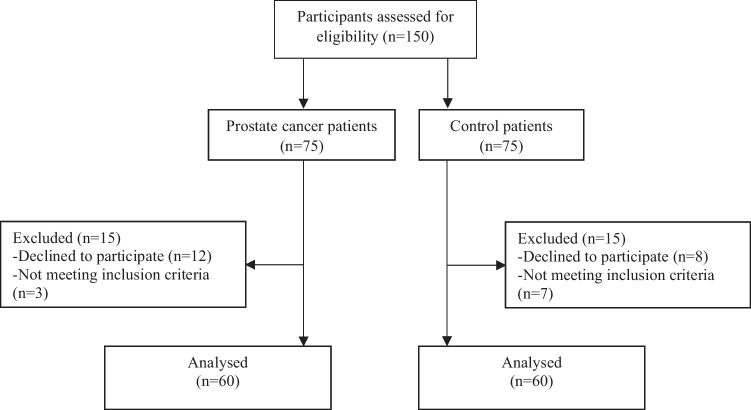
Flow diagram of participants

A total of 120 men, 60 prostate cancer survivors treated with radiotherapy and 60 aged-matched controls were finally included (Fig. 1). The characteristics of the study population are summarized in Table 1.

**Table 1:** Participants characteristics per group

Characteristic	Cancer patients (*n* = 60)	Control patients (*n* = 60)	*p* Value
Age (years ± SD)	61.23 ± 6.45	62.68 ± 4.87	0.954
BMI (mean ± SD)	27.89 ± 5.68	26.45 ± 10.72	0.671
Comorbidities (mean ± SD)	3.56 ± 1.14	2.96 ± 0.37	0.382
Cancer stage *n* (%)			
1	2 (3,33)	–	–
2	46 (76,66)	–	–
3	12 (20)	–	–
Treatment (%)			
Hormonal therapy	18 (30)	–	–
Chemotherapy	7 (11.66)	–	–
Surgery	11 (18.33)	–	–

Demographic characteristics were similar in both groups. The mean of comorbidities of the patients was similar in the two groups. The cancer survivors group presented with a higher BMI.

Of the sample, a diagnosis of stage II (76.66%) and stage III (20%) cancer was most commonly identified. In addition to radiotherapy, almost the entire sample indicated that some type of cancer-related treatment had been received, with hormonal therapy being the most reported (30%), followed by surgery (18.33%).

In Table 2, barriers and applied activity measures were presented per group. Regarding perception of benefits for physical activity and potential barriers, significant differences were also observed between groups with worse results in the cancer patients group for the benefits and barriers subscales and the overall score (*p* < 0.001). There were significant differences for the total physical activity levels (*p* = 0.018) with higher levels of physical activity in the control group.

**Table 2:** Barriers and applied activity measures per group

Variables	Cancer patients (*n* = 60)	Control patients (*n* = 60)	*p* value
Light activity subscore (IPAQ)	1434.09 ± 1699.90	1665.66 ± 3067.04	0.732
Moderate activity subscore (IPAQ)	243.98 ± 419.83	738.40 ± 948.93	0.020*
Vigorous activity subscore (IPAQ)	193.73 ± 657.10	1880.44 ± 3767.04	0.036*
IPAQ total	1869.29 ± 1715.50	4206.50 ± 4472.46	0.018*
Exercise benefits	95.31 ± 17.10	106 ± 9.87	*P* < 0.001*
Exercise barriers	30.62 ± 6,24	22.35 ± 5.31	*P* < 0.001*
EBBS total	139.67 ± 19.45	155.65 ± 11.88	*P* < 0.001*

*EBBS* Exercise Benefits/Barriers Scale, *IPAQ* International Physical Activity Questionnaire

*p < 0.05

In Table 3, quality of life and self-efficacy to manage chronic disease differences between groups are presented. Significant differences were found, the cancer patients group presented with poorer results in the following EQ-5D subscales: self-care (*p* = 0.045), usual activities (*p* < 0.001), pain/discomfort (*p* < 0.001), anxiety/depression (*p* = 0.026) and VAS (*p* < 0.001). Regarding self-efficacy, significant differences were also observed between groups (*p* = 0.040) with a greater score in the control group.

**Table 3:** Quality of life and self-efficacy measures per group

	Cancer patients (*n* = 60)	Control patients (*n* = 60)	*p* value
EQ-5D			
Mobility subscore	1.13 ± 0.34	1.08 ± 0.27	0.332
Self-care subscore	1.07 ± 0.25	1.00 ± 0.00	0.045*
Usual activities subscore	1.23 ± 0.43	1.02 ± 0.14	*p* < 0.001*
Pain/discomfort subscore	1.53 ± 0.70	1.06 ± 0.24	*p* < 0.001*
Anxiety/depression subscore	1.33 ± 0.48	1.15 ± 0.36	0.026*
VAS	73.75 ± 14.07	85.87 ± 11.62	*p* < 0.001*
Self-efficacy	54.19 ± 7.64	51.18 ± 6.88	0.040*

*EQ-5D* EuroQol-5 Dimension, *VAS* Visual Analogue Scale, *IPAQ* International Physical Activity Questionnaire, *SEMCD-S* Self-Efficacy to Manage Chronic Disease

*p < 0.05

## Discussion

This cross-sectional study aimed to measure self-reported PA levels of prostate cancer survivors after radiotherapy treatment, assess perceived barriers to PA in cancer survivors and determine quality of life and self-efficacy to manage chronic disease. Those aspects can be related to PA levels after a prostate cancer radiotherapy treatment. Findings of this study appear to suggest that self-reported PA levels after a radiotherapy treatment in prostate cancer survivors were lower than control age-matched men with similar body weight and height and presented more barriers to physical activity.

The population characteristics in our study is similar to other studies [44, 45]. Due to the fact that the mean age of the samples studied is representative of those who are candidates for radiotherapy. In addition, the inclusion and exclusion criteria of this study have the potential to eliminate people with older ages due to the greater likelihood that they present comorbidities that could significantly influence the study variables.

Diagnosis of prostate cancer usually led to undergo radiotherapy treatment. This treatment can substantially raise some impairments on health-related quality of life and associated lifestyles impacting current and future health of patients. In this line, prostate cancer patient profile needs to identify particularly concrete variables that can impact morbidity and mortality.

Regarding the first aim, our results revealed that self-reported PA level was lower in prostate cancer survivors after radiotherapy than control aged-matched men, thus agreeing with previous studies which showed that the proportion of prostate cancer patient who undertake regular exercise is low [18, 46, 47]. Despite the fact that the recommendations of the American College of Sports Medicine are 150 min (min) of moderate intensity or 75 min of vigorous physical activity per week to improve their overall health in cancer patients, prostate cancer survivors showed fewer minutes of moderate (*p* < 0.020) and vigorous (*p* < 0.036) physical activity than controls. In line with our results, Ozdemir K et al. [48] observed that only 20.7% of prostate patients in their study were physically active.

Our second aim was to analyze whether cancer survivors presented barriers and knew the benefits of PA. Our findings clearly demonstrate that prostate cancer patients after treatment presented more barriers and lower knowledge about benefits of PA than controls. Our study is in line to previous reviews [49, 50] that explored the influence of benefits and barriers of PA in prostate cancer survivors, the importance of understanding the characteristics of physical activity participation, the perceived barriers to exercise and the benefits of exercise are well known. These showed that the key facilitators to participation in PA include advice and guidance from healthcare professionals or specialists, avoiding the ‘rest-paradigm’ [51]. The study of Min J et al. [52] explored the relationship between PA levels and the most common barriers in prostate cancer, consistent with our results showing that prostate cancer patients present more of a barrier to activity than healthy controls. Our study shows that 1 year after diagnosis, prostate cancer patients remain inactive when compared to similar age and gender controls; this can be curious because control subjects have a similar number of comorbidities. One reason to those differences in PA levels between groups can be the information provided to subjects about the relevance of PA on their clinical profile; another reason can be the differences among major cancer survivor groups’ overall health behaviour. While a cancer diagnosis has been referred to as a possible ‘teachable moment’ where cancer patients can be more motivated to make lifestyle changes to improve health outcomes, the marker of physical activity has been reported to be under-considered among prostate cancer survivors in the long term after diagnosis [53].

The third aim was to determine quality of life and self-efficacy to manage chronic disease after a prostate cancer radiotherapy treatment. Despite quality of life has a large spectrum and numerous factors can condition the state estimate, low physical activity levels influenced negatively in quality of life [54]. Our results showed that prostate cancer survivors with low moderate and vigorous physical activity levels presented a worse self-perceived health status. Along the same lines, previous studies observed that prostate cancer survivors with higher PA levels are associated with better self-perceived quality of life [55–57]. Similarly, levels of self-efficacy were low in prostate cancer survivors. Mosher CE et al. [58] showed that self-efficacy plays an important role in PA and health promotion. The study of Yang R [59] et al. observed that information support program improved self-efficacy during oncological medical treatment; nevertheless, it is necessary to provide information support after coadjutant treatment.

### Study limitations

We must take into account some factors to properly interpret the results of the study. To begin with, as this is a cross-sectional study, and therefore cross-sectional data collection, it is impossible to establish a direction of causality. In addition, the number of participants was suggested to be sufficient to complete an adequate sample size; however, the individuals in the convenience sample consisted of only one region, which may influence the external validity of the results. Finally, the adjuvant treatment that patients received may have interfered with the results of the study. Concretely, hormone therapy can be of interest, but at long term, the possible impacts of those treatments have been reported as minimal [60, 61]. In another side, other authors have described no significant differences on clinical profile according to adjuvant treatments on prostate cancer at long term [62]. Even so, this is an aspect that may be relevant, and future studies comparing patients with hormone therapy added to radiotherapy and those without hormone therapy are necessary to contrast the results.

## Conclusion

In conclusion, the results of this study reveal that self-reported PA levels, as measured using the IPAQ, were low in prostate cancer survivors after treatment. Results also showed worse perception of benefits for PA and potential barriers by the cancer survivors. Similarly, the quality of life and self-efficacy to manage chronic disease of prostate cancer survivors was lower. These results sustenance the need to design intervention programs focusing on these outcomes.

## Data Availability

The data that support the findings of this study are available upon request from the corresponding author.
